# Equine Herpesvirus 1 Variant and New Marker for Epidemiologic Surveillance, Europe, 2021

**DOI:** 10.3201/eid2710.210704

**Published:** 2021-10

**Authors:** Gabrielle Sutton, Camille Normand, Flora Carnet, Anne Couroucé, Marie Garvey, Sophie Castagnet, Christine I. Fortier, Erika S. Hue, Christel Marcillaud-Pitel, Loïc Legrand, Romain Paillot, Pierre-Hugues Pitel, Ann Cullinane, Stéphane Pronost

**Affiliations:** University of Caen Normandy, Caen, France (G. Sutton, C. Normand, F. Carnet, A. Couroucé, C.I. Fortier, E.S. Hue, L. Legrand, S. Pronost);; LABÉO Frank Duncombe, Saint-Contest, France (G. Sutton, C. Normand, F. Carnet, S. Castagnet, C.I. Fortier, E.S. Hue, L. Legrand, R. Paillot, P.-H. Pitel, S. Pronost);; Cisco-Oniris, Nantes, France (A. Couroucé);; Irish Equine Centre, Johnstown, Naas, Ireland (M. Garvey, A. Cullinane);; Réseau d’Épidémio-Surveillance en Pathologie Équine, Saint-Contest (C. Marcillaud-Pitel, L. Legrand, P.-H. Pitel, S. Pronost);; Writtle University College, Chelmsford, UK (R. Paillot)

**Keywords:** equine herpesvirus 1, viruses, Valencia, Spain, Europe

## Abstract

Equine herpesvirus 1 isolates from a 2021 outbreak of neurologic disease in Europe have a mutation, A713G, in open reading frame 11 not detected in 249 other sequences from equine herpesvirus 1 isolates. This single-nucleotide polymorphism could help identify horses infected with the virus strain linked to this outbreak.

Equine herpesvirus 1 (EHV-1) is a threat to the equine industry, as demonstrated by the ongoing outbreak of neurologic disease initially reported at a large equestrian event in Valencia, Spain. EHV-1 infection is associated with respiratory disease, abortion in mares, neonatal death of foals, ocular disease, and, more rarely, encephalomyelopathy. As of March 26, 2021, a total of 18 horses had died during the outbreak: 11 in Spain, 5 in Germany, and 2 in Belgium. As the horses have returned from Spain to their training yards, the virus has spread to 9 other countries in Europe and to Qatar.

EHV-1 is endemic in horse populations worldwide. Reactivation of latent virus can occur at any time, but infected horses are more vulnerable when exposed to stress. When an outbreak occurs during an equestrian event and horses return to their respective countries or regions, the emergence of new cases of EHV-1 in the weeks and months after often elicits questions regarding the involvement of the strain from the original outbreak.

A total of 850 horses from many different countries were in attendance at the CES Valencia Spring Tour competition in February 2021; of those, 180 horses stayed in Valencia after the venue was closed by authorities in Spain. Most of these horses were pyrexic, and some developed neurologic disease. Nasopharyngeal swab samples were collected from 67 horses and sent to LABÉO Frank Duncombe (Saint-Contest, France) for PCR analysis. We tested 19 positive samples by the allelic-discrimination real-time PCR for a single-nucleotide polymorphism (SNP), A2254G, within open reading frame (ORF) 30, which has been associated with the neuropathogenic phenotype of EHV-1 (*1*). However, this association is not absolute, and the 19 samples tested positive for the A2254 genotype (i.e., the genotype more commonly associated with the nonneuropathogenic phenotype of EHV-1).

Viruses (FR/Valencia1/2021 and FR/Valencia2/2021) from 2 horses from France that remained in Valencia were isolated on cell culture and characterized by multilocus sequence typing (MLST) as belonging to clade 10 (*2*–*4*). MLST demonstrated that the virus associated with the outbreak of encephalomyelopathy in Valencia was closely related to other viruses circulating for several years in Europe; however, this tool is not accurate enough to identify a specific strain. Analysis of the sequences of the different ORF fragments used for the MLST revealed a mutation at position 713 of ORF11 (A713G) in FR/Valencia1/2021 and FR/Valencia2/2021 when compared with reference strains Ab4 and V592. This A713G mutation was not identified in 103 ORF11 sequences obtained in GenBank from strains isolated in the United Kingdom, United States, China, Australia, Belgium, New Zealand, Japan, or India. Furthermore, this mutation was not identified in 131 ORF11 sequences from strains isolated in Ireland or in 15 ORF11 sequences from EHV-1 strains isolated in France. Although we cannot exclude the existence of this mutation in other strains from the field, its absence in the 249 ORF11 sequences analyzed suggests that this SNP constitutes a possible marker to identify horses infected with the virus strain linked to the Valencia outbreak.

Because the Federation Equestre Internationale (Lausanne, Switzerland), the international governing body of equestrian sports, cancelled international events in 11 countries in Europe during March 1–April 11, 2021, the detection of this SNP might be helpful for investigating the extent of virus spread in different countries. For example, the identification of this ORF11 A713G genotype in a nasopharyngeal swab specimen taken from a horse with no known link to the event in Valencia triggered further investigations. These investigations revealed that the horse had stayed in a stable that, just a day before, had lodged a sick horse that had returned from Valencia.

Further investigations are warranted to determine the role of this mutation, which induces a change of lysine to arginine (K238R) in ORF11 (tegument protein). This SNP is helpful for differentiating EHV-1 cases linked to the recent epizootic in Spain from the many other strains of EHV-1 circulating in different countries. It might also help identify, in the future, abortions linked to the Valencia strain. A sensitive real-time PCR for the routine detection of this variant in clinical samples is under development and will be extremely useful in tracking the virus and in performing molecular epidemiology studies at the European level. Unlike MLST or whole-genome sequencing, such an assay could be readily deployed in a diagnostic laboratory. 
